# A Cross-Sectional Survey to Assess Biorisk Management System in Research and Diagnostic Laboratories in Khyber Pakhtunkhwa, Pakistan

**DOI:** 10.3389/fpubh.2021.766162

**Published:** 2021-12-17

**Authors:** Javed Muhammad, Samreen Sarwar, Tariq Khan, Shamsul Arfin Qasmi, Aamer Ikram, Ghufran Ahmad, Maria Zahid, Rida Haroon Durrani, Furqan Ahmed

**Affiliations:** ^1^Department of Microbiology, University of Haripur, Haripur, Pakistan; ^2^Department of Microbiology, University of Health Sciences Lahore and Technical Advisor, Health Security Partners, Lahore, Pakistan; ^3^Department of Biotechnology, University of Malakand, Chakdara, Pakistan; ^4^Department of Pathology/Microbiology, Fazaia Ruth Pfau Medical College, Karachi, Pakistan; ^5^National Institute of Health, Islamabad, Pakistan; ^6^NUST Business School, National University of Sciences and Technology (NUST), Islamabad, Pakistan; ^7^Molecular Pathology Department, Dow University of Health Sciences, Karachi, Pakistan; ^8^University Diagnostic Laboratory, Central Laboratory Complex, the University of Veterinary and Animal Sciences, Pattoki, Pakistan; ^9^Department of Prevention and Evaluation, Leibniz Institute for Prevention Research and Epidemiology, Bremen, Germany

**Keywords:** biosafety, laboratories, Khyber Pakhtunkhwa, Pakistan, biorisk management

## Abstract

Financial, cultural, and managerial hurdles have made biosafety and biosecurity measures difficult in resource-constrained countries like Pakistan. Because of increasing awareness of biorisk management, diagnostic and research laboratories have made major advances in biosafety and biosecurity in the recent decade. As a result, identifying and addressing gaps in biorisk management has never been more critical. The purpose of this study was to assess the current situation of personal protective equipment (PPE), biosafety behavior, waste management, biosafety and biosecurity measures, training and safety, and health services in diagnostic and research laboratories across Pakistan's Khyber Pakhtunkhwa (KP) province. We adapted the WHO Laboratory Assessment tool (2012) and CWA 15793 (Biorisk management guidelines) for conducting a cross-sectional survey, which was distributed among various laboratories in KP. The survey included 30 laboratories, including 11 diagnostic and 19 research laboratories. In comparison to diagnostic laboratories, biorisk management practices in research laboratories were better in terms of PPE, biosafety behavior, waste management, biosafety measures, biosecurity measures, trainings, and safety and health services. KP laboratories' biorisk management practices have improved over time, according to our findings. However, we were able to identify inadequacies that would require considerable improvements to the current setups based on the WHO and CWA 15793 recommendations. Organizations can tailor their biosafety measures and training to address identified gaps using the presented KP snapshot.

## Introduction

To avoid laboratory-acquired infections and control the spread of potentially hazardous agents in the environment, diagnostic and research laboratories must maintain a safe and secure environment (1). For safe and secure practices, laboratories must have a complete Biorisk Management (BRM) system that complies with the Global Health Security Agenda (GHSA) and bioethical guidelines (1–3).

Laboratory BRM has been given a high priority, especially among scientific circles, throughout the world for the past few decades (4). Numerous advancements in biosafety and biosecurity practices and procedures have emerged from this level of prioritization. Furthermore, through systematic awareness and capacity building, this has led to progress in the use of equipment and administrative controls, particularly in developed regions of the world (5). Despite the increased number of laboratory research and diagnostic settings in low and middle-income countries (LMICs), progress has been gradual (4–6).

Despite limited and inadequate funding allocated to BRM, Pakistan has made significant progress as a result of national and international organizations' efforts to raise awareness and build capacity. In Pakistan, however, public health, scientific research, veterinary medicine, and diagnostic laboratories face administrative and financial challenges. Pakistan currently has a number of challenges, including a strain on the health-care system due to its large population, a scarcity of health-care professionals, particularly in rural areas, a lack of oversight mechanisms, and limited resources allocated to improving or maintaining safe healthcare practices (7). Leadership and administration in many clinical and research settings in Pakistan are struggling to prioritize BRM due to an already overburdened healthcare system.

KP is Pakistan's third most populous province with a population of 30.52 million (8). KP hosts 11 private and 30 public universities and research institutes, 277 hospitals, and a number of diagnostic and biomedical facilities (9). In comparison to other provinces, a study conducted in KP in 2012 found that improper use of personal protective equipment (PPE), lack of proper sharps disposal mechanism, lack of standard operating procedure for laboratories, and accident reporting systems were the highest (9). Since 2012, a number of national and international organizations, as well as the Pakistani government, have been striving to build BRM capability and raise awareness in compliance with the GHSA and International Health Regulations (IHR) (8–11). These efforts have sensitized many stakeholders, including diagnostic laboratories, research institutions, and academics in taking responsibility and prioritizing BRM at their laboratory settings in Pakistan.

Since 2012, no survey for evaluating BRM systems in KP laboratories has been conducted. Furthermore, the 2012 study only examined only diagnostic or hospital settings (9). As a result, the purpose of this survey was to assess BRM systems in diagnostic and research laboratories in KP province in order to better identify the gaps and opportunities for future research and capacity-building efforts (9).

## Materials and Methods

For assessing and appraising laboratory BRM systems, a variety of tools and guidelines are available (12, 13). The questionnaire was developed in accordance with CWA 15793 (Biorisk management guidelines) and the WHO Laboratory Assessment Tool (2012) for evaluating BRM systems in KP laboratories for this study (14, 15). Both approaches have been utilized in a variety of settings. They cover a wide range of biosafety and biosecurity indicators, as well as practices and procedures, behaviors, safety and health services, waste disposal, and the use of personal protective equipment (PPE). The cross-sectional survey was conducted using an online questionnaire (12–15). The survey was conducted from September through November of 2016. Laboratory technicians, technologists, supervisors, quality control managers, postgraduate students, research officers, and faculty from universities, diagnostic, and research laboratories were the target respondents. Since we aimed to include institutes rather than individuals, convenience-based sampling was used to identify and recruit respondents for the survey. There were two components to the survey questionnaire. The first section of the questionnaire inquired about the type of laboratory and the respondents' titles and affiliations. The second section included questions about compliance and resource availability in the domains of PPE, safety and security procedures, behaviors, training, waste disposal protocol implementation, and health service information. Table 1 includes all the categories, variables, and questions included in the survey. All aspects assessed in these laboratories were given codes from Variable 1 (V1) to Variable 54 (V54).

**Table 1:** The list of variables (V) used for the cross-sectional survey to assess biorisk management system in research and diagnostic laboratories in Khyber Pakhtunkhwa, Pakistan.

**Indicator**	**V**	**Indicator**	**V**	**Indicator**	**Variable**
Lab coat	v1	Use of liquid disinfection	v21	Eyewash station	v41
Gloves	v2	Implementation status of liquid disinfection	v22	Emergency Shower	v42
Goggles	v3	Methods to ensure the efficacy of disinfection	v23	Does your staff/students have access to workers health services?	v43
Where are coats and lab linens washed?	v4	Are procedures for safe and secure transport of culture, specimens, samples, and other contaminated materials effectively?	v24	Does your staff/students follow a regular yearly visit to workers health services?	v44
Is protective clothing of approved design and fabric provided for all staff/students for everyday work?	v5	Are the biosafety procedures available at the bench level?	v25	Are individuals considered unfit for work on health grounds identified and prevented from accessing areas where there are risks of exposure?	v45
PPE for Chemical and radiation	v6	Do you use biosafety cabinets to manipulate samples producing potentially dangerous aerosols?	v26	Are conditions that could impact personnel associated with the facility addressed? These may include medical conditions affecting work, the ability to use appropriate PPE safely, or factors affecting general well-being	v46
Face Shield	v7	Do you have a biohazard sign indicated on the doors of the rooms where microorganisms are handled?	v27	Have the vaccination needs been identified?	v47
Are staff prohibited from wearing the protective clothing outside of the lab?	v8	Are warning and accident prevention signs used to minimize work hazards?	v28	Is there an immunization program for the lab?	v48
Are staff prohibited from wearing open-toed footwear?	v9	Are areas requiring vaccinations to enter indicated?	v29	Are women of childbearing age warned of the consequences of working with certain microorganisms, carcinogens, mutagens, and teratogens?	v49
Are staff prohibited from eating, drinking, smoke, or apply cosmetics in the lab working areas?	v10	Are controls in place to ensure that demand originates from legitimate facilities and individuals?	v30	Are women of childbearing age told that if they are, or suspect that they are, pregnant, they should inform the appropriate medical/scientific staff member so that alternative working arrangements may be made for them if necessary?	v50
Is it prohibited to store food or drinks in the lab working areas?	v11	Is access to lab areas restricted to authorized personnel?	v31	Are first-aid boxes provided at strategic locations?	v51
Is mouth pipetting forbidden?	v12	Is the whole building securely locked when unoccupied?	v32	Are qualified first-aiders available?	v52
Do you have separate disposals for infectious and non-infectious wastes?	v13	Are rooms containing hazardous materials and expensive equipment locked when unoccupied?	v33	Are such first-aiders trained to deal with emergencies peculiar to the lab, e.g., contact with corrosive chemicals, accidental ingestion of poisons and infectious materials?	v53
Do you have covered waste disposal containers?	v14	Is access to such rooms, equipment and materials appropriately controlled and documented?	v34	Are notices prominently posted giving clear information about first-aiders' location, telephone numbers of emergency services, etc.?	v54
Do you have safe and adapted waste containers?	v15	Have the staff/students been presented with a biosafety manual?	v35		
Do you have special sharps containers?	v16	Is training on “Biosafety while sampling” required for your lab staff/students before work?	v36		
Do you have dedicated waste for used solvents?	v17	Is training on “Using disinfectants and procedures in disinfection” required for your lab staff/student before work?	v37		
Have all potential waste streams and other sources of contamination been identified and documented?	v18	Is training on “Proper waste management” required for your lab staff/students before work?	v38		
Is there an adequate organization for the collection and disposal of general household rubbish?	v19	Are refresher training on these topics organized at least every 3 years?	v39		
Are discarded infectious materials removed daily or more often and disposed of safely?	v20	Were lab workers, e.g., domestic and clerical staff, instructed on the lab's potential hazards and the material it handles?	v40		

### Ethics Statement

According to approval from the Departmental Bioethics Committee, Department of Microbiology, Hazara University, Mansehra, Pakistan with letter number F.No.HU/MB/BEC/2016/10-05, informed consent was acquired from study participants, and respondents were informed that their participation in the survey was voluntary. No personal information was linked to the data acquired during analysis, and all responses were kept anonymous and confidential.

### Statistical Analysis

SPSS 20.0 was used to analyze the data, and Microsoft Excel was used to generate the graphs. Depending on whether the laboratory was diagnostic or research-based, we segregated our results. PPE, biosafety behaviors, waste management, biosafety measures, biosecurity measures, training, and safety and health services were divided into seven groups for further stratification (Table 1).

## Results

### Participant Details

A total of 30 laboratories from KP responded to the online survey, including 11 diagnostic and 19 research laboratories. The respondents belonged to Swabi, Peshawar, Haripur, Mardan, Nowshehra, Mansehra, Kohat, Bannu, Swat, DI Khan, Dir regions of KP.

### Personal Protective Equipment

**Figure 1:**
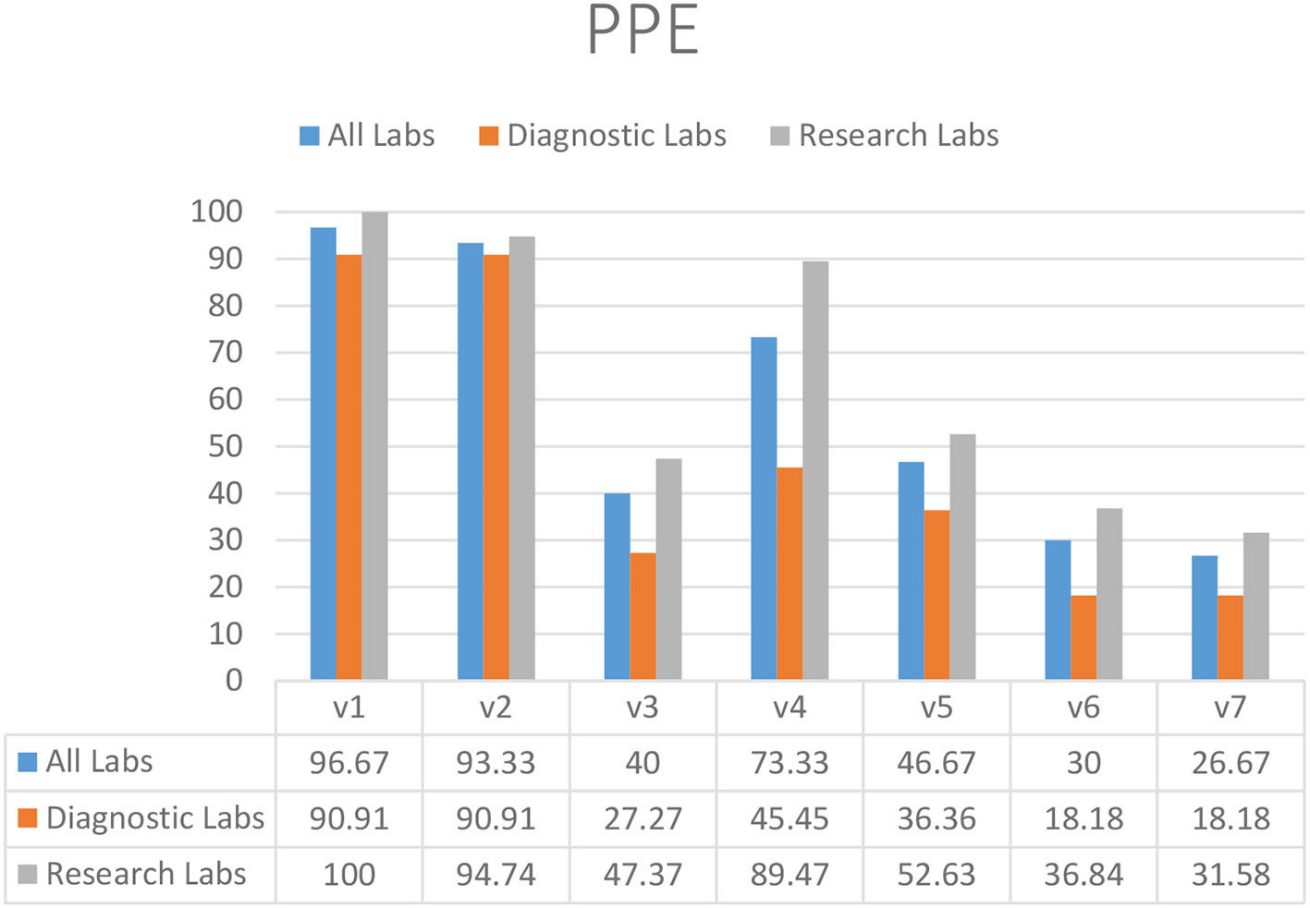
Availability and appropriate usage of PPE in diagnostic and research laboratories in Khyber Pakhtunkhwa (KP), Pakistan.

The majority of laboratories used gloves (93.33%) (V2) and lab coats (96.67%) (V1), although diagnostic laboratories demonstrated reduced compliance with the guideline that lab coats should not be washed at home (73.33%) (V4) (Figure 1). Face shields (26.67%) (V7), goggles (40.00%) (V3), clothing of approved design and fabric (46.67%) (V5), and PPE for chemical and radiation protection (30.00%) (V6) were used and available in limited laboratories in KP (Figure 1).

### Biosafety Behaviors

**Figure 2:**
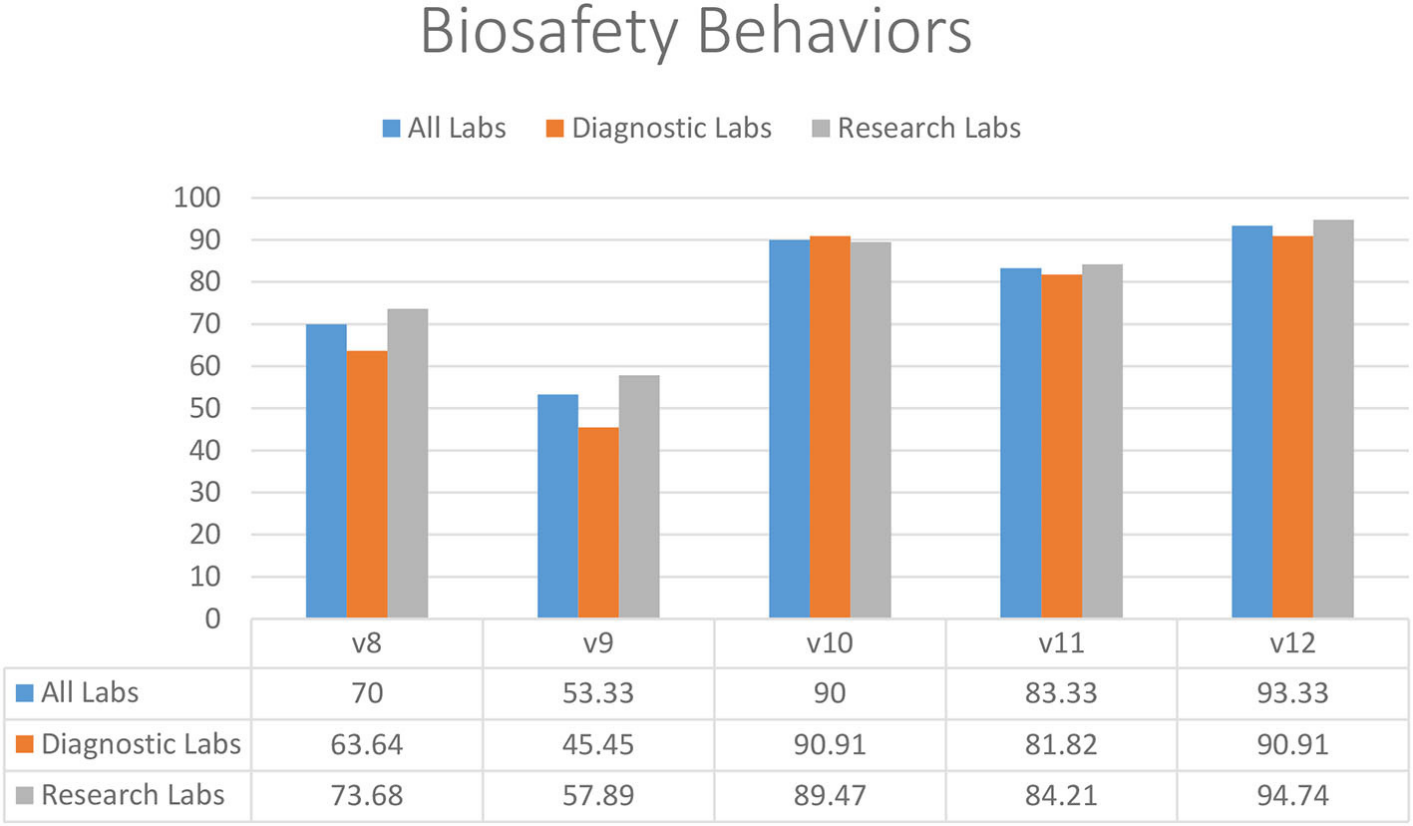
Biosafety behaviors in diagnostic and research laboratories in Khyber Pakhtunkhwa (KP), Pakistan.

In the restrictions on food storage inside the laboratory (83.33%) (V11), eating and drinking in the working area (90.00%) (V10), and mouth pipetting (93.33%) (V12), laboratories demonstrated substantial compliance (Figure 2). Almost half of diagnostic and research laboratories did not have a protocol in place to reduce or limit the use of open footwear (53.33%) (V9) in the lab (Figure 2). In addition, there was significantly less compliance with the restriction on wearing protective clothing outside of laboratories (70.00%) (V8) (Figure 2).

### Waste Management

**Figure 3:**
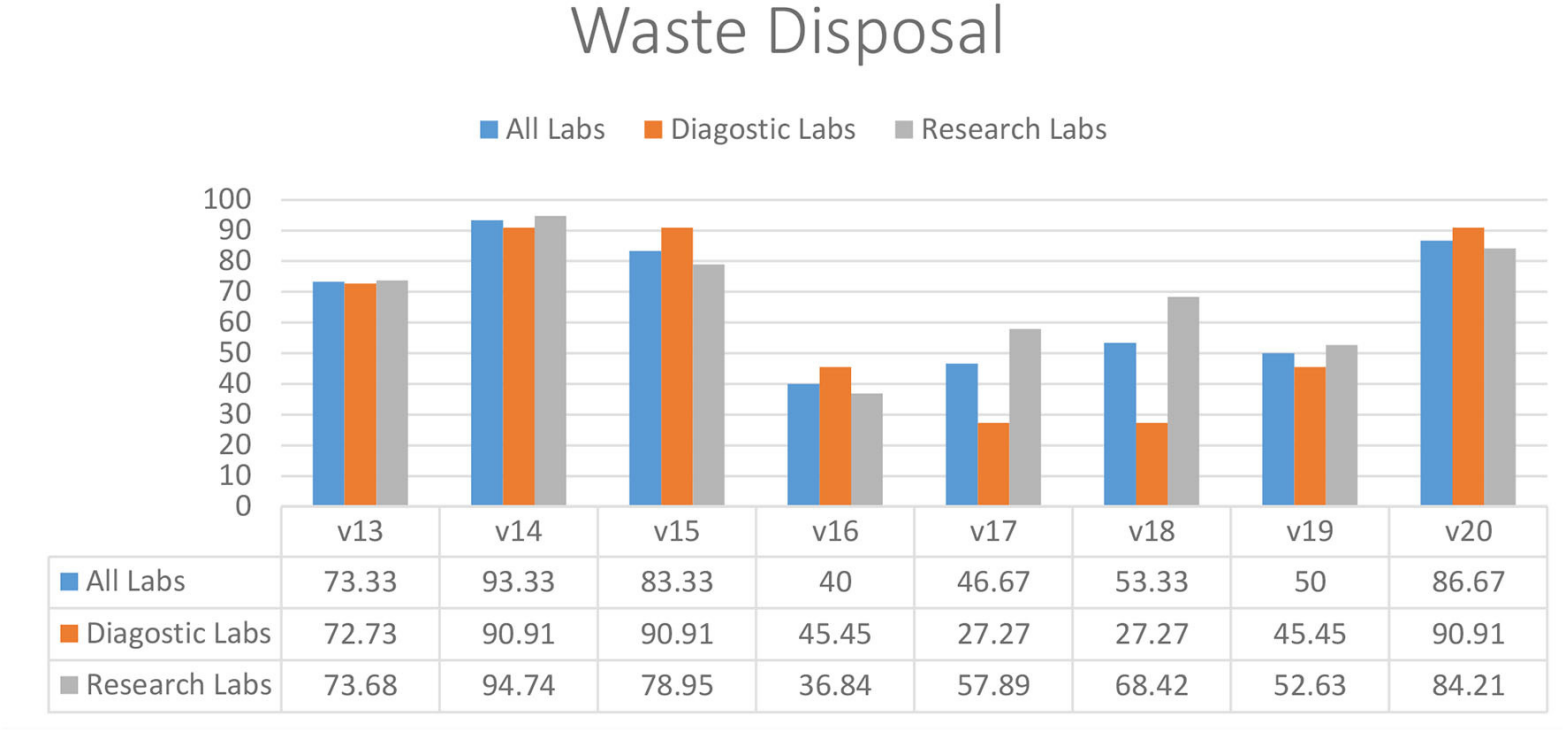
Waste management practices in diagnostic and research laboratories in Khyber Pakhtunkhwa (KP), Pakistan.

Most of the diagnostic and research laboratories had separate disposal containers for infectious and non-infectious waste (73.33%) (V13), excluding sharp containers (93.33%) (V14) and biological waste containers available (83.33%) (V15) (Figure 3). Almost half of the diagnostic (45.45%) and research (36.84%) labs did not have a dedicated sharps container available (V16). Discarded infectious materials were removed daily or more often in most laboratories (86.67%) (V20). Diagnostic laboratories were struggling with having dedicated waste for used solvents (27.27%) (V17) and identifying all potential waste streams (27.27%) (V18). Most of the diagnostic and research laboratories also did not have an adequate organization for collecting and disposing of household rubbish (50.00%) (V19).

### Biosafety Measures

**Figure 4:**
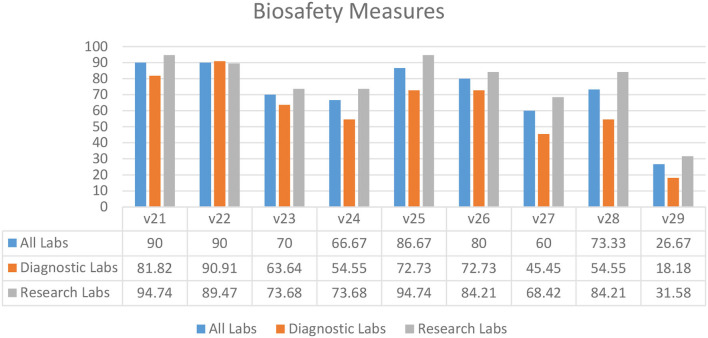
Biosafety measures in diagnostic and research laboratories in Khyber Pakhtunkhwa (KP), Pakistan.

According to the survey results, the majority of diagnostic and research laboratories in KP were compliant in the use of liquid disinfectants (90.00%) (V21), implementation of liquid disinfection (90.00%) (V22), written biosafety procedures available at the bench (86.67%) (V25), use of biosafety cabinets for aerosol-generating procedures (80.00%) (V26), and display of accident prevention signs (73.33%) (V28) (Figure 4). Several laboratories lacked indications of areas requiring vaccination (26.67%) (V29), implementation of safe and secure sample transport (66.67%) (V24), and display of biohazard signs on the doors of rooms where microorganisms are handled (60.00%) (V27) (Figure 4).

### Biosecurity Measures

**Figure 5:**
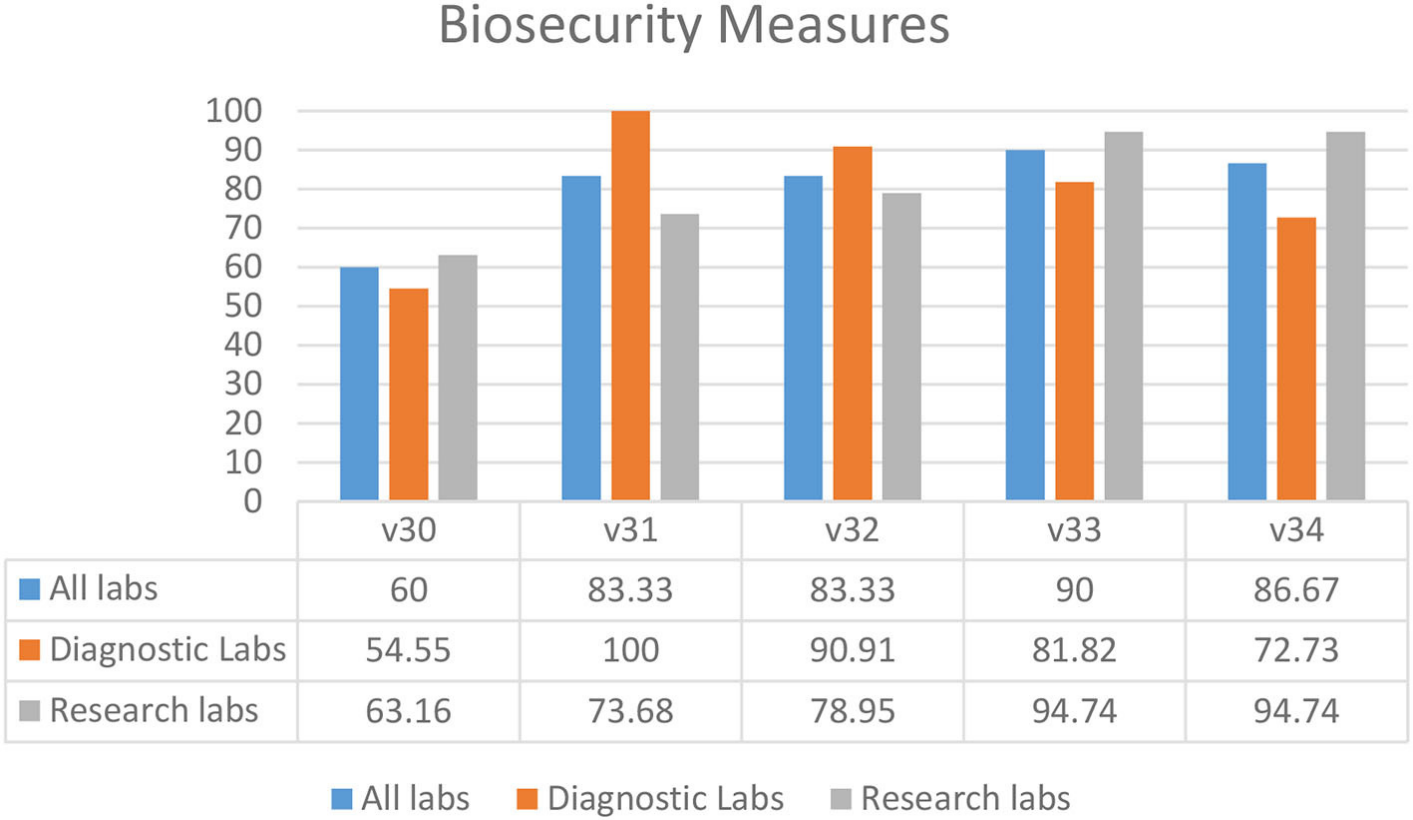
Biosecurity measures in diagnostic and research laboratories in Khyber Pakhtunkhwa (KP), Pakistan.

Overall, the results showed that biosecurity measures were being followed in laboratories throughout the KP province (Figure 5). In most laboratories, access and security of laboratory settings (V31–34) were deemed adequate. “Controls in place to ensure demand originates from legitimate facilities or individuals” (60.00%) (V30) was the most undermined biosecurity practice. The overall percentage of biosecurity controls and measures compliance (80.67%) in KP province shows a positive picture in both research and diagnostic settings (Figure 5).

### Training

**Figure 6:**
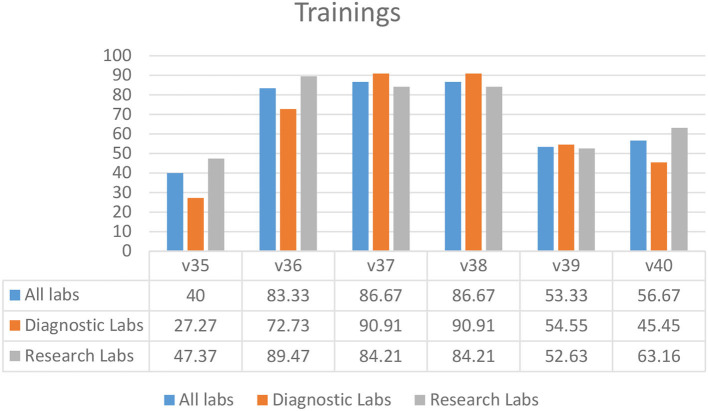
Training practices in diagnostic and research laboratories in Khyber Pakhtunkhwa (KP), Pakistan.

Most laboratories required biosafety training for students and staff prior to sampling (83.33%) (V36), the use of disinfectants (86.67%) (V37), and proper waste management (86.67%) (V38) (Figure 6). Fewer laboratories had mandatory 3-year refresher training (53.33%) (V39) and training for auxiliary staff (56.67%) (V40) (Figure 6). A biosafety manual was not available to 60.00% of the laboratory staff and students (V35) (Figure 6).

### Safety and Health Services

**Figure 7:**
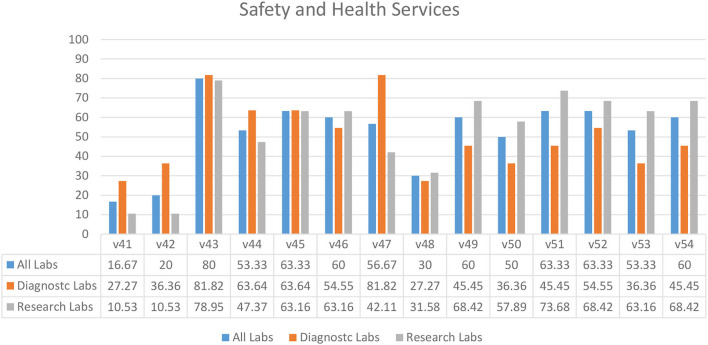
Safety and health services in diagnostic and research laboratories in Khyber Pakhtunkhwa (KP), Pakistan.

Survey results indicated that laboratories had an ineffective immunization program (30.00%) (V48) in their facilities (Figure 7). Diagnostic laboratories had better compliance for identifying the needs for vaccination (81.82%) (V47) and an annual visit to health services by staff members (63.64%) (V44), as compared to the research laboratories (Figure 7). This compliance might be due to the affiliation of most diagnostic laboratories with hospital settings. In almost half of the laboratories, access to first aid boxes (63.33%) (V51) and qualified first aiders were missing (V52–53). A similar pattern was seen for safety and health variables relevant to pregnancy and childbearing age while working in a laboratory (V49–50) (Figure 7). Most of the laboratories did not have an eyewash station (16.67%) (V41).

## Discussion

### Diagnostic Laboratories Comparison: 2012 and 2016

**Figure 8:**
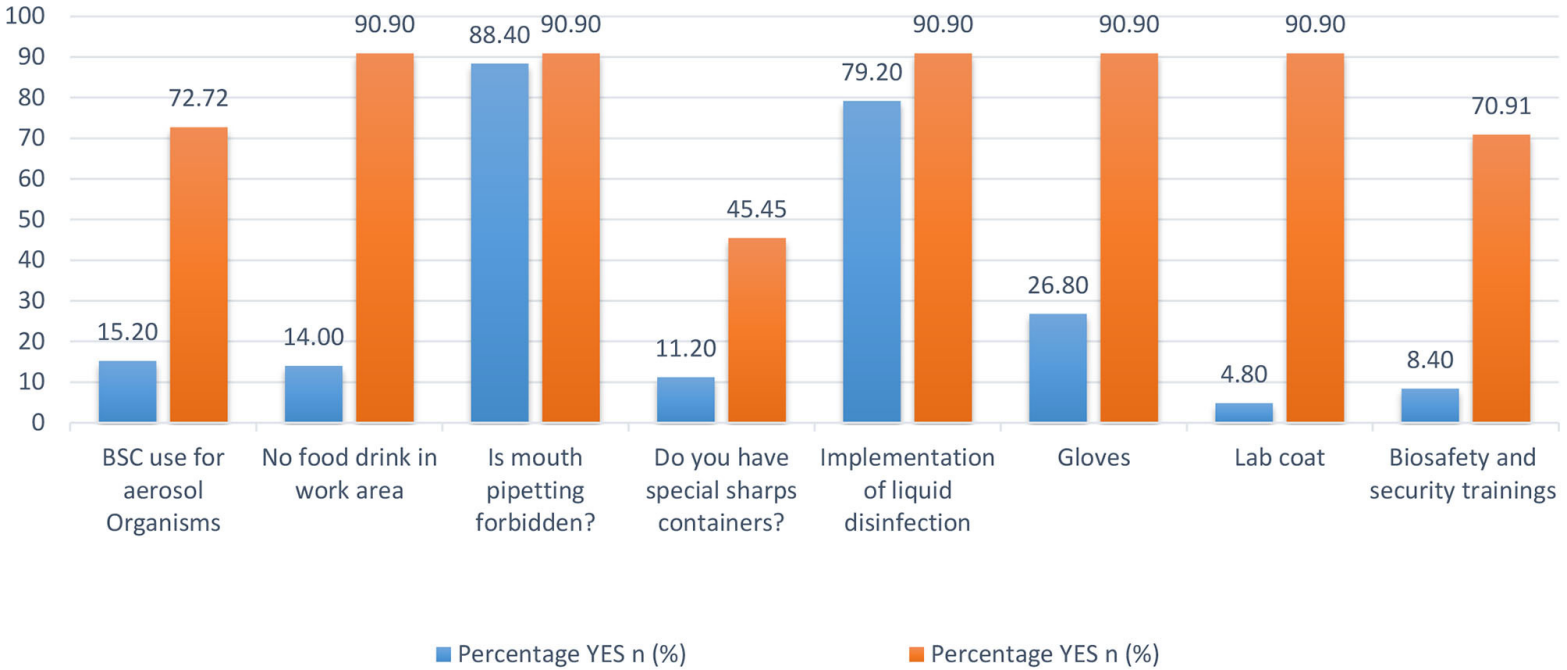
Biorisk management system variables' comparison between Khyber Pakhtunkhwa (KP) diagnostic laboratories in 2012 and 2016.

In 2012, a cross-sectional survey in Pakistan evaluated BRM systems in diagnostic settings (9). Nasim et al. created a diagnostic laboratory questionnaire that included questions about routine laboratory practices, mouth pipetting, PPE, disinfection methods, and specimen handling and collection. We compared our 2016 data to the results of the survey conducted in 2012 (9) to assess the current state of BRM systems in diagnostic laboratories and any progress made over time (Figure 8). We found eight standard variables in both data sets. When the data was compared, all eight variables show significant improvements (9). Despite the low level of sharps container compliance in KP (45.4%) in 2016, there has been a significant improvement from 11.2 percent of diagnostic laboratories in 2012. Since 2012, the use of biosafety cabinets, the absence of food and drink in the work area, the availability of gloves and lab coats, and biosafety and security training have all improved significantly. Many national and international organizations have been working with Pakistani laboratories to improve BRM systems in recent years, and this significant improvement can be attributed to them.

In 2018, another study looked at the impact of training on BRM practices at five universities in one of KP's districts (16). According to Rashid and colleagues, 82 percent of the students had received BRM training and were found to have the knowledge and skills to properly use PPE, manage waste, and respond to emergencies (15). Rashid et al., on the other hand, found a significantly lower compliance rate in some universities, indicating the need for additional interventions to put the knowledge and skills learned during these trainings into practice. Further research into the reasons for resource constraints and low leadership engagement and priority toward BRM should be investigated to identify specific factors impeding implementation (15, 16).

A 2017 study in KP assessed compliance with hospital waste management rules in 44 public and private hospitals, uncovering serious shortcomings in the hospital waste management systems (1). However, when compared to the previous study, our findings revealed significant improvements in the waste management system in KP laboratories (9). This disparity could be explained by the sample investigated, as we were looking at waste management in laboratories rather than hospitals. Some significant deficiencies in the laboratories' health and safety services were found during our investigation. In these laboratories, a robust occupational health and medical/incident surveillance program should be prioritized for long-term improvement and evaluation (9).

### Follow-Up Activities and Progress Related to the Improvement of Biorisk Management System Across Institutions

Since 2014, the Pakistan Biological Safety Association (PBSA) has collaborated with the Fogarty International Center on the BioPrism flagship program to develop biosafety practices in Pakistan. The program employed a three-tiered training-of-trainers approach. Sixty professionals are being taught the fundamentals of biosafety from all over the country. Pre- and post-tests are used to assess their understanding of the concepts as well as the training's effectiveness. At the end of training, each participant is asked to demonstrate the skills they gained. Top achievers were selected to participate in a 5-day “master trainer” course to improve their presentation and communication skills. Verbal exams were conducted after the master trainer course to assess the trainers' comprehension of the subject, delivery, and communication skills. Each trainer is assigned a topic to present, and their skills were assessed depending on how successfully they do so. High scorers were then selected for a third, more intensive “wet workshop.” At the completion of the session, the high achievers were given the title of master trainer. All participants, including master trainers, should first train at least seven individuals and report to PBSA. These trainings were successful in establishing a network of dedicated and well-trained biosafety professionals. PBSA has launched a new series of workshops in Pakistan called Responsible Conduct in the Life Sciences. Participants should train at least seven people and report to the PBSA, including master trainers. In the same way that the BioPrism program prepares participants to become trainers, these seminars do as well (17). Multiple workshops on high-reliability organization, influence without authority, and waste management were held at the national and regional levels by PBSA and FIC/NIH in conjunction with biorisk management experts. In addition, the program's trainers have been offered support in conducting training in their individual institutions to promote biorisk management principles (17).

## Conclusion

The laboratories in KP are evidently working hard to improve their BRM systems and practices, as indicated by this study. These efforts must be reinforced, with a focus on continuous improvement, which is critical for successful BRM systems. Continual improvement necessitates thorough inspections and audits of BRM systems to identify non-conformities. This study provides an overview of the current BRM systems' strengths and areas for improvement. Despite the fact that leadership engagement has become so vital in this process, more research is needed to determine how to gain public sector leadership to invest and prioritize BRM for continued improvement.

## Data Availability Statement

The original contributions presented in the study are included in the article/supplementary material, further inquiries can be directed to the corresponding author/s.

## Ethics Statement

The studies involving human participants were reviewed and approved by Departmental Bioethics Committee, Department of Microbiology, Hazara University, Mansehra, Pakistan with letter number F.No.HU/MB/BEC/2016/10-05. The patients/participants provided their written informed consent to participate in this study.

## Author Contributions

JM: conceptualization, methodology, writing, and review and editing. SS: conceptualization, methodology, project administration, and review and editing. SQ, AI, and TK: critical review. GA: statistical analysis and interpretation. MZ and RD: writing the first draft of results and discussion. FA: writing of the original draft, statistical analysis and interpretation of the data, and supervision. All authors contributed to the article and approved the submitted version.

## Conflict of Interest

The authors declare that the research was conducted in the absence of any commercial or financial relationships that could be construed as a potential conflict of interest.

## Publisher's Note

All claims expressed in this article are solely those of the authors and do not necessarily represent those of their affiliated organizations, or those of the publisher, the editors and the reviewers. Any product that may be evaluated in this article, or claim that may be made by its manufacturer, is not guaranteed or endorsed by the publisher.
